# Rectal synovial sarcoma case report – An unexpected cause of acute lower gastrointestinal bleeding

**DOI:** 10.1016/j.ijscr.2024.110613

**Published:** 2024-11-15

**Authors:** Aldara Faria, Daniel Jordão, Alberto Figueira, Teresa Pereira, Carlos Ferreira

**Affiliations:** Department of General Surgery, Hospital de Santa Maria, Unidade Local de Saúde de Santa Maria, Departamento de Cirurgia Geral, Unidade de Saúde Local de Santa Maria, Avenida Professor Egas Moniz MB, 1649-028 Lisbon, Portugal; Faculty of Medicine of the University of Lisbon, Faculdade de Medicina da Universidade de Lisboa, Avenida Professor Egas Moniz MB, 1649-028 Lisboa, Portugal

**Keywords:** Synovial sarcoma, Soft tissue sarcomas, Gastrointestinal bleeding

## Abstract

**Introduction and importance:**

Acute lower gastrointestinal bleeding is one of the most common causes of hospital admission. However, massive bleeding is uncommon and is mainly due to hemorrhoidal bleeding in elderly patients receiving anticoagulant therapy. We present a rare case of a massive rectal haemorrhage with an uncommon cause.

**Case presentation:**

A 60-year-old woman was admitted to the emergency department with a two-day history of lower gastrointestinal bleeding. During digital rectal examination and anoscopy, a palpable mass located 8 cm from the anal verge with severe bleeding was detected. Subsequent rectosigmoidoscopy revealed a bulge in the rectal wall with mucosal ulceration and signs of recent bleeding. The CT-scan revealed a 74 × 41 mm locally advanced rectal mass and three hepatic lesions (segments 6 and 7). Pathology results were compatible with synovial sarcoma (SyS). The case was discussed by a sarcoma board, and the patient underwent doxorubicin plus ifosfamide resulting in a significant reduction of the metastatic lesions and complete remission of the primary lesion on MRI. Following multidisciplinary discussion, low anterior resection of the rectum with terminal colostomy and right posterior sectionectomy were performed. At 23 months follow-up, the patient shows no signs of recurrence.

**Clinical discussion:**

SyS are uncommon malignant tumours, accounting for 5–10 % of all soft tissue sarcomas. Metastatic SyS carries a poor prognosis.

**Conclusion:**

Gastrointestinal involvement is exceptionally rare and, to the best of our knowledge, this is the third rectal SyS case reported in the literature. Because of this, SyS should be managed in sarcoma referral centers.

## Highlights

• Soft tissue sarcomas (STS) are uncommon solid tumours of mesenchymal origin, comprising for 1 % of all adult malignancies.
• Synovial sarcoma accounts for 5–10 % of all STS.
• Synovial sarcoma as a cause of lower acute gastrointestinal bleeding is extremely rare.
• A combination of surgery plus chemotherapy could be an option in fit patients.

## 1 Introduction and importance

Soft tissue sarcomas (STS) are uncommon solid tumours of mesenchymal origin, comprising for 1 % of all adult malignancies [1]^,^ [2]. Synovial sarcoma (SyS) is the third most common type of STS [1], accounting for 5–10 % of all STS. These lesions can arise anywhere in the body, but gastrointestinal involvement is extremely rare, with cases associated with metastases being even rarer [[3], [4], [5], [6]]. Here, we present a case of a 60-year-old woman with acute lower intestinal bleeding stemming from a SyS of the rectum. To the best of our knowledge this is the third case of rectal SyS reported in international literature [4,7]. This work is reported in line with SCARE statement criteria. [8]

## 2 Case presentation

A 60-year-old woman was admitted to the emergency department with two days history of persistent lower gastrointestinal bleeding and no other symptoms. She has no known exposure to carcinogens or drugs and no relevant family or psychosocial history. Her medical and surgical history was unremarkable. Digital rectal examination and anoscopy revealed abundant rectal bleeding, leading to tamponade. Despite this, the patient experienced progressive hemorrhagic shock. She was taken to the operating room for a rectosigmoidoscopy which revealed an extramucosal lesion protruding into the rectum, located 8 to 10 cm from anal verge, with ulceration and signs of recent bleeding (Fig. 2). Biopsies were performed. A subsequent contrast enhanced-CT scan showed a locally advanced rectal lesion with 74 × 41 mm (Fig. 3), along with three hepatic lesions (segments 6 and 7) suggestive of metastasis (Fig. 4). Pathologic analysis confirmed a rectal SyS with the presence of SYT-SSX-fusion in molecular studies.

**Fig. 1:**
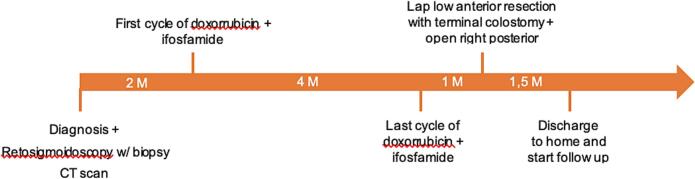
Timetable of the events. M, months, w/, with, Lap, laparoscopic.

The case was discussed by a sarcoma board and systemic chemotherapy was proposed. She underwent five cycles of doxorubicin and ifosfamide. During this period, she received 25Gy of local hemostatic radiotherapy. Upon re-evaluation by CT scan and abdominal and pelvic MRI, a complete local response rate was observed, along with a reduction in secondary hepatic lesions. Further multidisciplinary discussion led to the decision to proceed with a laparoscopic low anterior resection with terminal colostomy and a right posterior sectionectomy via a Kocher incision, both performed by a consultant. The postoperative course was complicated by: 1) biliary leak, which was managed with percutaneous drainage (Clavien-Dindo classification 3a), and 2) evisceration of the small intestine through the stoma site (Fig. 5), requiring surgical reintervention to reduce the content and a mesh placement (Clavien-Dindo classification 3b). The second postoperative course was uneventful. Fig. 1 shows the timetable of the sequence of events.

**Fig. 2:**
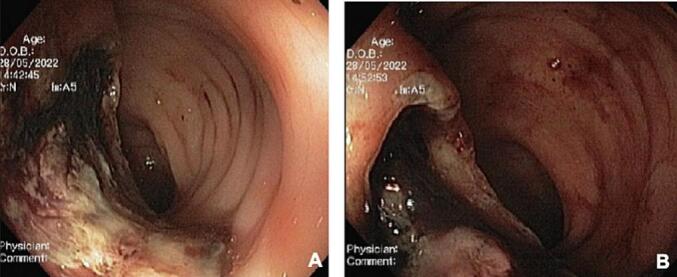
Mucosal ulceration by a protruding mass (A) and signs of recent bleeding (B).

**Fig. 3:**
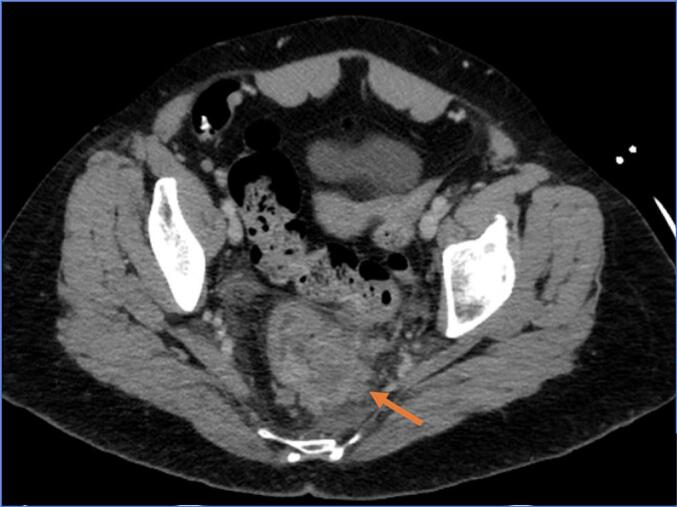
Rectal tumour on CT scan (arrow).

**Fig. 4:**
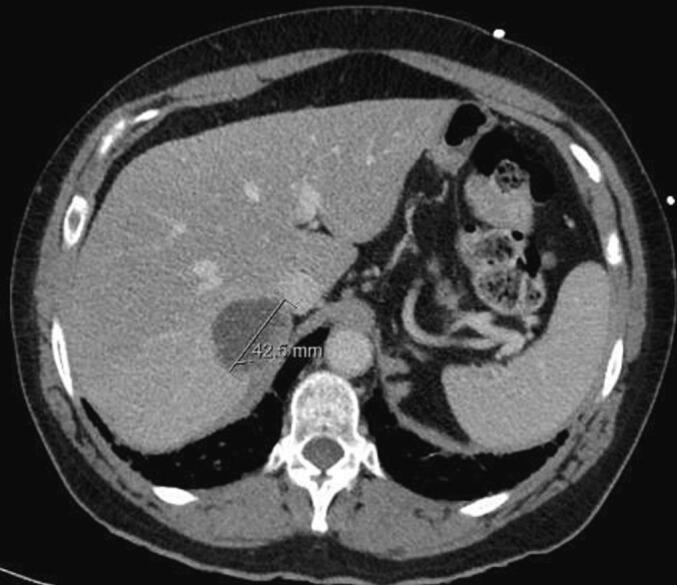
Hepatic metastasis.

**Fig. 5:**
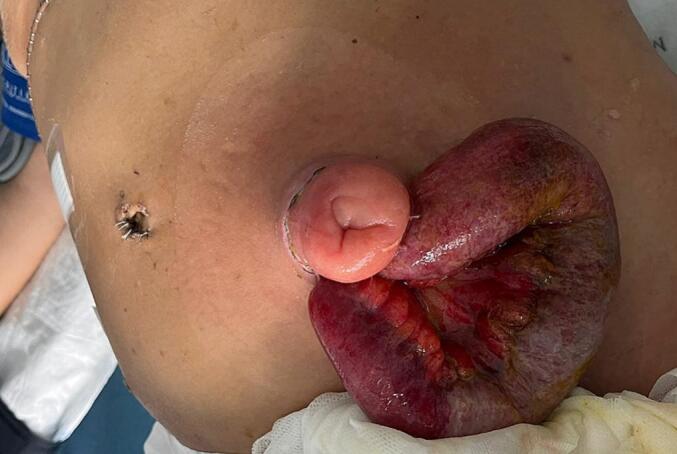
Evisceration from stoma orifice.

Final pathologic analysis showed a complete pathologic response in both the rectum and liver (ypT0N0M0). Currently, at one year and eleven months of follow-up, there are no signs of relapse.

## 3 Clinical discussion

Synovial sarcoma (SyS) is a rare malignant tumour, accounting for 5–10 % of all soft tissue sarcomas [9]. Most patients are diagnosed with localised disease, but 6–18 % of patients have synchronous metastasis [9]. Late metastasis incidence is high, reaching 50–70 %, with the lung being the most common site (80 %), followed by bone (9,9 %) and liver (4,5 %) [10].

The characteristic translocation between chromosomes X and 18, t(X;18), resulting in an SYT-SSX-fusion, is pathognomonic. This oncogenic fusion protein impacts cellular transcription and metabolism, leading to sarcomagenesis [[10], [11], [12], [13]].

SyS is most commonly affects the lower limbs of young adults [14] and typically presents as a slow-growing tumour [1,15] with no specific symptoms [9]. Gastrointestinal SyS, as in our case, is extremely rare, with the rectum reported as the tumour site in only three cases in the international literature [3,4,7]. The diagnostic gold standard is MRI, which typically reveals a heterogeneous, multi-lobed soft tissue mass with T2-weighted features (“triple sign”), internal haemorrhage, and calcifications [1]. An image-guided core needle biopsy is required to differentiate SyS from other STS [15]. In our case, due to severe haemorrhage, biopsies were performed in the operating room and through rectosigmoidoscopy.

After diagnosis and staging, it is important to distinguish the localised from advanced disease. Surgical resection with wide excision ensuring negative margins, combined with radiotherapy (neoadjuvant or adjuvant), is the mainstay of treatment. However, despite this, current ESMO guidelines also recommend three cycles of neoadjuvant full-dose anthracycline plus ifosfamide chemotherapy [9]. Optimal treatment for metastatic cancer is not well established [16]. For fit patients with single organ, resectable disease, surgery or local radiotherapy can be considered, with positive postoperative survival outcomes [9,16]. In adult patients with advanced or metastatic SyS, the gold standard systemic treatment is doxorubicin plus ifosfamide, which achieved a response rate of 60 % [9].

The prognosis is dismal in metastatic SyS, with a 3-year overall survival of 27 % [10]. However, cohort studies comparing survival rates between patients undergoing metastasectomy at non-pulmonary sites and those who did not show consistent survival benefits for the former group [16].

In our case, given that the patient was healthy and without significant comorbidities, we opted to perform the resection of the primary tumour and hepatic metastases, achieving excellent outcomes so far, with no evidence of recurrence.

## 4 Conclusion

Gastrointestinal SyS is extremely rare, and cases with metastatic components carry a very poor prognosis. Current treatment strategies for metastatic SyS rely on cytotoxic therapies. In highly selected, fit patients, a treatment approach combining resection of the primary tumour, metastasectomy of a single affected organ, and systemic chemotherapy may be considered reasonable, as it has shown improved prognosis.

## Informed consent

Written informed consent was obtained from the patient for publication and any accompanying images. A copy of the written consent is available for review by the Editor-in-Chief of this journal on request.

## Ethical approval

The reported case is a clinical case, with consent provided by the patient, and does not constitute clinical research from the institution.

## Funding

This research did not receive any specific grant from funding agencies in the public, commercial or not-for-profit sectors.

## CRediT authorship contribution statement

Aldara Faria, Daniel Jordão – study design, data collection, data analysis and interpretation and writing paper.

Alberto Figueira, Teresa Pereira, Carlos Ferreira– data interpretation and corrections.

## Guarantor

The guarantor of the case report is Aldara Faria.

## Declaration of competing interest

The authors declare they have no conflict of interest.
